# Evaluation of Strategies to Fight COVID-19: The French Paradigm

**DOI:** 10.3390/jcm10132942

**Published:** 2021-06-30

**Authors:** Audrey Giraud-Gatineau, Philippe Gautret, Philippe Colson, Hervé Chaudet, Didier Raoult

**Affiliations:** 1 IHU Méditerranée Infection, 13005 Marseille, France; audrey.giraud.gatineau@gmail.com (A.G.-G.); philippe.gautret@ap-hm.fr (P.G.); philippe.colson@univ-amu.fr (P.C.); herve.chaudet@univ-amu.fr (H.C.); 2 Institut de Recherche pour le Développement (IRD), Assistance Publique-Hôpitaux de Marseille (AP-HM), Service de Santé des Armées (SSA), Vecteurs—Infections Tropicales et Méditerranéennes (VITROME), Aix Marseille University, 13005 Marseille, France; 3 French Armed Forces Center for Epidemiology and Public Health (CESPA), Service de Santé des Armées (SSA), 13014 Marseille, France; 4 Assistance Publique-Hôpitaux de Marseille (AP-HM), 13005 Marseille, France; 5 Institut de Recherche pour le Développement (IRD), Assistance Publique-Hôpitaux de Marseille (AP-HM), Microbes Evolution Phylogeny and Infections (MEPHI), Aix Marseille University, 13005 Marseille, France

**Keywords:** COVID-19, SARS-CoV-2, France, deaths, Marseille, Paris

## Abstract

(1) Background: We collected COVID-19 mortality data and the age distribution of the deceased in France and other European countries, as well as specifically in the cities of Paris and Marseille, and compared them. (2) Methods: Data on mortality related to COVID-19 and the associated age distribution were collected from government institutions in various European countries. In France, data were obtained from INSEE and Santé Publique France. All-cause mortality was also examined in order to study potential excess mortality using EuroMOMO. The Marseille data came from the epidemiological surveillance system. (3) Results: France is one of the European countries most impacted by COVID-19. Its proportion of deaths in people under 60 years of age is higher (6.5%) than that of Italy (4.6%) or Spain (4.7%). Excess mortality (5% more deaths) was also observed. Ile-de-France and the Grand-Est are the two French regions with the highest mortality. The proportion of deaths in the under-60 age group was considerable in Ile-de-France (9.9% vs. 4.5% in the Southern region). There are significantly higher numbers of patients hospitalized, in intensive care and deceased in Paris than in Marseille. (4) Conclusions: No patient management, i.e., from screening to diagnosis, including biological assessment and clinical examination, likely explains the high mortality associated with COVID-19.

## 1. Introduction

In December 2019, a novel coronavirus known as SARS-CoV-2 (severe acute respiratory syndrome coronavirus 2) emerged and spread from Hubei Province, China, to the rest of the world in a few months. The disease (COVID-19) did not spare any continent and was declared a pandemic by the WHO on 11 March 2020. As of 11 June 2021, 174,977,063 cases of COVID-19 and 3,773,600 deaths related to this disease had been reported worldwide [1]. The epidemic seemed to be diminishing or even stopping in Western European countries in June 2020, as has been observed in Asia, but an upsurge in the number of cases was observed in early July 2020 when borders were reopened.

The management of COVID-19 has been subject to considerable divergences around the world. These divergences have concerned containment measures, the systematization of virus detection tests, isolation and therapeutic strategies. The same is true within France. Indeed, to limit the spread of the virus, the French government initially decided to close schools and universities [2] as well as all cultural facilities, such as theatres and museums, and cancelled large gatherings of people [3]. A total lockdown was finally decreed on 17 March 2020 with the objective of stopping the chain of transmission [4]. However, unlike in Iceland or Korea, no mass screening was then carried out systematically on a national scale in the early stage of this pandemic, which would have made it possible to quickly obtain information on the incidence of the disease and thus put in place public health measures better adapted to the particularities of the spread of the virus in our territory [4,5,6]. Only “individuals with clinical signs of acute respiratory infection with documented or subjective fever who have travelled to or stayed in a high-risk exposure area within 14 days prior to the date of onset of clinical signs, or individuals who have had close contacts with a confirmed COVID-19 case or any person showing signs of pneumonia or acute respiratory distress” were screened in March 2020 [7]. These positive patients were quarantined for 14 days. Finally, mass screening was proposed on 11 May 2020, when lockdown was lifted, and borders reopened.

In our hospital institute in Marseille, France, organized mass screening was carried out beginning 27 January 2020, comparable to what was implemented in Iceland, and hydroxychloroquine/azithromycin combined therapy (HCQ + AZ) was proposed in our institute for most COVID-19 patients [8]. In Paris and the Ile-de-France region, early treatment was not proposed, nor was any therapy that was not officially approved until the end of May and the lockdown [9].

The case fatality due to COVID-19 is a key point of the epidemic situation and provides insights into the management and outcome of COVID-19 according to the health policies in place at the time of the study. We were interested in comparing the mortality in European countries during the first period (March–June 2020). We paid special attention to the mortality by age, which appeared to us to be an important marker. Indeed, mortality in people over 80 years of age is usually high in the winter and summer periods in temperate countries due to the circulation of common respiratory viruses. Besides, it is possible that mortality in people under 60 years of age can serve as a marker of the effectiveness of therapeutic management in a given situation. Then, we focused on France and regional disparities, and finally in two major French cities, Paris and Marseille. These comparisons at several levels allowed us to discuss strategies involving only social measures versus both social and medical measures.

## 2. Materials and Methods

### 2.1. Governmental Policies against COVID-19

The number of SARS-CoV-2 tests per 1000 inhabitants was obtained using data from the aggregator site Our World in Data as of 2 June 2020 [10].

In order to study the severity of governments’ responses to the epidemic, an overall government response Severity Index, which is a composite measure based on 9 indicators including school closures, workplace closures and travel bans, reduced to a value between 0 and 100 (100 = strictest response), was retrieved from Oxford COVID-19 Government Response Tracker and Blavatnik School of Government [11].

Information on lockdown policy was also collected.

### 2.2. COVID-Related Mortality in France and Other European Countries

The mortality associated with COVID-19 in France and different European countries during the first wave of the COVID-19 spread was collected from the Center for Systems Science and Engineering (CSSE) at Johns Hopkins University [1] between January 2020 and 2 June 2020. To correct the biases linked to the size of the countries, we calculated the mortality rate per million inhabitants. The case fatality rate (CFR, the ratio of the number of deaths to the number of confirmed cases, i.e., the lethality rate) was obtained from the aggregator site Our World in Data as of 2 June 2020. The age distribution of individuals who died from COVID-19, when available, was collected for several countries. Data for the United Kingdom were collected from the National Health Service (NHS) (https://www.england.nhs.uk/statistics/statistical-work-areas/covid-19-daily-deaths/) as of 2 June those for Italy were collected via Epicentro, Istituto Superiore di Sanità (https://www.epicentro.iss.it/) as of 1 June; those for Spain were collected via El Centro de Epidemiologia (CNE) (https://cnecovid.isciii.es/) as of 29 May; and those for Germany were collected via INED (https://dc-covid.site.ined.fr/en/data/germany/) by 2 June. The proportion of deaths among people under 60 years of age was calculated. The mortality aboard 3 ships, namely, the Diamond Princess, the Roosevelt and the Charles de Gaulle, has also been documented in the literature [12,13,14].

### 2.3. Excess All-Cause Mortality in France and Other European Countries

All-cause mortality in France and at the regional level according to the decedents’ place of residence between 1 January and 31 August for 3 years (2018, 2019 and 2020) was collected from the INSEE database [15] to assess potential excess mortality. Excess mortality in Paris and Marseille was also studied using the same database.

The excess mortality by country and by age group (0–14, 15–44 and 45–65 years) was retrieved from the EuroMOMO website (https://www.euromomo.eu/graphs-and-maps, accessed on 11 June 2021), which collects all-cause mortality data from several European partner countries such as France, the United Kingdom, Italy and Spain. The excess mortality was estimated using the z-score, which allows comparisons of mortality between the different countries and the different time periods studied [16].

### 2.4. French Departmental and Regional Data on COVID-19

Daily hospital data related to COVID-19 by French region and department were obtained from Santé Publique France, a public health institute in France [17]. These data were available only from 19 March onward, and our study period was therefore limited to 19 March to 2 June 2020. To correct for biases related to population size in each region and department, we calculated the mortality rate in France per million inhabitants. The size of each regional population was retrieved from the Institut National d’Études Démographiques (INED) database [18]. The population of each department was collected from the INSEE database.

Deaths associated with COVID-19 by age group according to region were retrieved from GEODES, the French Public Health mapping observatory, over the same study period [19].

### 2.5. Seroprevalences in French Regions and Probability of Mortality

Adjusted estimates of seroprevalences in Ile-de-France, in the Grand-Est, and in New-Aquitaine were collected in an article preprinted on MedRxiv in September 2020 [20]. The seroprevalence in the Bouches-du-Rhône is based on data from the Blood Establishment and is still unpublished. The overall probability of death among the infected cases was collected in the study of Salje et al., 2020, and equals 0.5% [21].

### 2.6. Marseille Data

Local data for the city of Marseille were obtained by using the epidemiological surveillance system from our institute that collects information on patients hospitalized at Assistance Publique-Hôpitaux de Marseille (AP-HM), which comprises four public university hospitals [22,23]. This system is based on the results from the clinical microbiology laboratory of the IHU Méditerranée Infection, and includes microbiological results (sample type, sample date, requesting unit) and anonymous patient information (age, sex, home postal code, date of admission), stored in a data warehouse using MariaDB. COVID-19-associated mortality data were obtained from the Department of Medical Information (DMI) of AP-HM.

The size of the Marseille population was also obtained from the INSEE database [24].

These data were collected from 27 January to 31 May 2020.

### 2.7. Statistical Analysis

The statistical analyses were performed with OpenEpi software (https://www.openepi.com/TwobyTwo/TwobyTwo.htm?fbclid=IwAR0NjbfgL6G7d77LiFSYTzdJAbK3YIPaYi2ZDFEeCnhFqbHFuMfibs1jaWI) (accessed on 11 June 2021). A chi-square test or mid-P test was used to compare groups, depending on the variables. The graphs were created using the software R [25] with the ggplot2 package [26] and using Excel.

## 3. Results

### 3.1. Government Policies against COVID-19

As for the number of tests per 1000 inhabitants, Iceland was the country that tested the most with 179.0 tests per 1000 inhabitants, followed by Denmark with 91.3 tests per inhabitants (Table 1). However, these two countries did not have strict lockdown measures and did not have a high Severity Index compared to other European countries (53.7 and 72.2, respectively).

Italy was the country that pursued the strictest and most severe policy to control the epidemic (Severity Index = 93.5), followed by France (Severity Index = 90.7) and Spain (Severity Index = 85.2). In contrast, these three countries had not implemented a mass screening policy.

Sweden is the country with the lowest Severity Index, with a number of tests per 1000 inhabitants 7.5 times lower than that of Iceland, which has a comparable Severity Index (Severity Index = 53.7).

### 3.2. Overall Analysis and Positioning of France in Relation to Other European Countries

**Figure 1:**
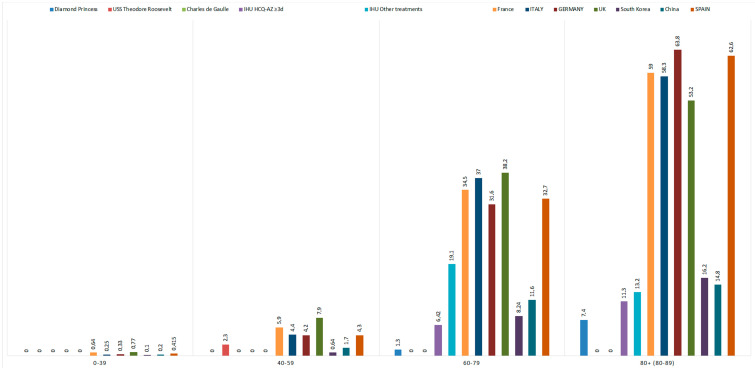
Mortality rate by age group on 3 ships (the Diamond Princess, the USS Theodore Roosevelt and the Charles de Gaulle) and in France, Italy, Germany, the United Kingdom (UK), Korea, China, Spain and our institute following treatment (hydroxychloroquine and azithromycin or other) [27].

France, with 28,836 deaths, is one of the countries most impacted by COVID-19 in Europe. Its mortality rate was 441.8 deaths per million inhabitants, placing it behind Belgium (825.4), Spain (580.2), the United Kingdom (576.6) and Italy (553.6). However, its case fatality rate (CFR) was 20%, the highest in Europe. France is also one of the countries where the proportion of deceased individuals under 60 years of age was especially high (6.5%, *n* = 1201), exceeding the rates in Italy (4.6%, *n* = 1487), Spain (4.7%, *n* = 969) and Germany (4.5%, *n* = 378) (Figure 1). The United Kingdom reached 8.6% (*n* = 2336). There were no deaths on the French warship Charles de Gaulle. One death (2.3%) of a 41-year-old man was recorded on the USS Theodore Roosevelt. On the Diamond Princess, a cruise ship, the majority of those who died were over 80 years of age (7.4%). Overall, Belgium, France, Italy, the Netherlands, Spain, Sweden and the United Kingdom had excess mortality at least 10 standard deviations above the mean. For the 45–65 age group, this excess mortality was highest for England (z-score of 25.9), followed by Spain (16.5) and France (8.8).

### 3.3. Comparison of Excess All-Cause Mortality

In France overall, 430,254 deaths due to all causes were observed between 1 January 2020 and 31 August 2020. This corresponds to an increase in the number of deaths by 4.6% and 5.0% compared to 2018 and 2019, respectively, for the corresponding periods of time (Table 2). The excess mortality was high in dependent elderly residents in retirement homes and at home but was not noted among hospitalized patients. Ile-de-France had the highest excess all-cause mortality (+22%) nationwide, while a decrease was observed in New-Aquitaine (Table 1). In Ile-de-France, the highest excess mortality was observed in residential facilities for elderly dependents and in hospices (+48% compared to 2018 and +55.3% compared to 2019). Excess mortality at home was approximately 28% in Ile-de-France, 14% in Grand-Est, 6% in Sud and 2% in New-Aquitaine. However, hospital mortality has decreased in Sud (approximately −6%) and New-Aquitaine (approximately −4%), while it has increased in Ile-de-France and Grand-Est by 15% and 6%, respectively. Regardless of the location of death, April had the highest excess mortality, with an increase of 32.7% over 2018 and 36.1% over 2019. A lower excess mortality of 17% (compared to 2019) was visible as early as March, particularly for Grand-Est. There was no significant increase or decrease in the other months studied (Supplementary Data S1–S4).

### 3.4. Mortality Rates Associated with COVID-19 in France

**Figure 2:**
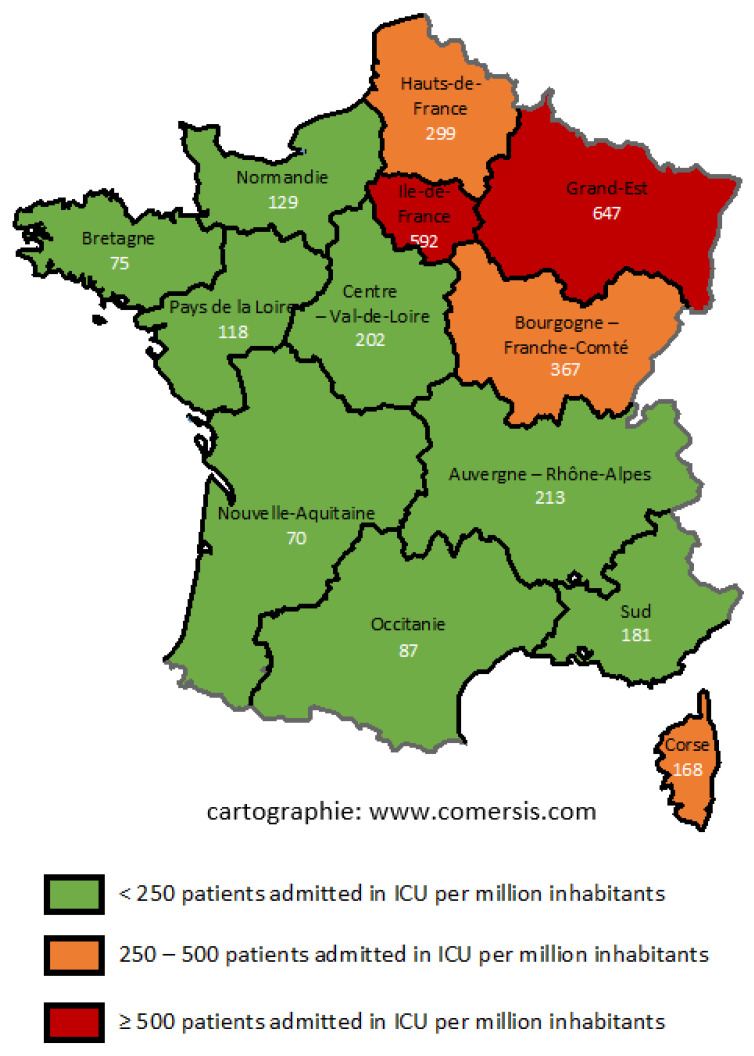
Regional distribution of COVID-19 mortality per million inhabitants. Green indicates regions that were less impacted by COVID-19, orange indicates regions that were moderately impacted by COVID-19 and red indicates regions that were strongly impacted by COVID-19. Map was obtained from www.comersis.com.

Three groups seemed to emerge according to mortality rates. Grand-Est and Ile-de-France were the two regions most impacted by COVID-19, with 647 and 592 deaths per million inhabitants, respectively (Figure 2). However, this rate was significantly higher in Grand-Est than in Ile-de-France (*p*-value < 0.001). Haut-Rhin, a department in the Grand-Est region, had the highest mortality rate, with 1095 deaths per million inhabitants. Within the Ile-de-France region, Paris was the most impacted department, with 798.9 COVID-19-related deaths per million inhabitants. Bourgogne-Franche-Comté and Hauts-de-France (regions bordering the two previous regions) had lower incidence rates, as did the island of Corsica, with 367,299 and 168 deaths per million inhabitants, respectively (*p*-value < 0.001). The other regions, including the Sud region (formerly known as the Provence-Alpes-Côte d’Azur region) (181 deaths per million inhabitants) and New-Aquitaine (70 deaths per million inhabitants), had mortality rates almost 3 times lower than that of the Grand-Est region. The Bouches-du-Rhône, a department in the Sud region where our institute is located, had a mortality rate of 263 deaths per million inhabitants.

### 3.5. Mortality Associated with COVID-19 in People under 60 Years of Age

COVID-19 killed mainly patients over 80 years of age. The 80–89 age group appeared to be the most impacted age group nationally, regionally and locally (Supplementary Table S1).

**Figure 3:**
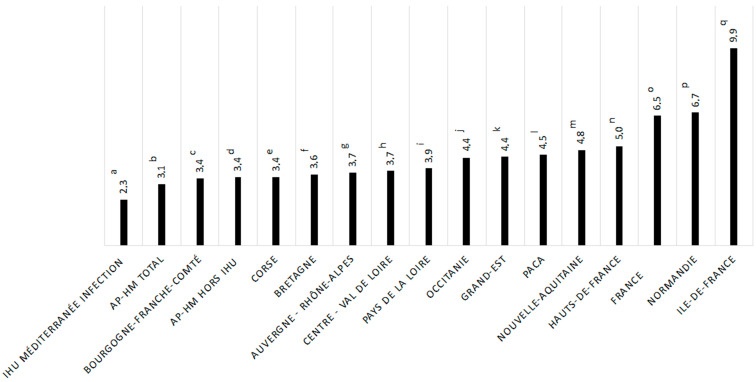
Comparison of mortality associated with COVID-19 in patients under 60 years of age at IHU Méditerranée Infection, at AP-HM, in French regions and in France overall. For comparing the mortality in patients under 60 years of age between regions, a statistical test was done: q vs. k, *p*-value < 0.001, q vs. i, *p*-value < 0.001, q vs. g, *p*-value < 0.001, q vs. b, *p*-value = 0.007, q vs. o, *p*-value < 0.001. ^a^, IHU Méditerranée Infection; ^b^, AP-HM total; ^c^, Bourgogne–Franche-Comté; ^d^, AP-HM without IHU; ^e^, Corse; ^f^, Bretagne; ^g^, Auvergen–Rhône-Alpes; ^h^, Centre–Val de Loire; ^i^, Paus de la Loire; ^j^, Occitanie; ^k^, Grand-Est; ^l^, PACA; ^m^, Nouvelle-Aquitaine; ^n^, Hauts-de-France; ^o^, France; ^p^, Normandie; ^q^, Ile-de-France.

Mortality rates in patients under 60 years of age varied according to region (Figure 3). This percentage reached 9.9% (*n* = 701) in the Ile-De-France region, which was significantly higher than that observed in the Sud region (4.5%, *p*-value = 0.0000003), in the Grand-Est region (4.4%, *p*-value ≤ 0.0000001), in the Auvergne-Rhône-Alpes region (3.7%, *p*-value ≤ 0.0000001) and in France overall (6.5%, *p*-value ≤ 0.00000001). No significant difference was observed regarding mortality in patients under 60 years among the Sud region (4.5%), the Auvergne-Rhône-Alpes region (3.7%) and the Grand-Est region (4.4%) (Supplementary Table S2).

### 3.6. Mortality Estimation According to the Prevalence of Antibodies Tested after the First Outbreak

In the Ile-de-France region as well as in the Grand-Est region, an excess of deaths could be observed compared to what was expected (592 deaths per million inhabitants instead of 500 in the Ile-de-France region and 647 deaths per million inhabitants instead of 450 in the Grand-Est region) (Table 3). Conversely, the Sud and New-Aquitaine regions had lower mortality per million inhabitants than expected.

### 3.7. Focus on Two French Cities: Paris and Marseille

**Figure 4:**
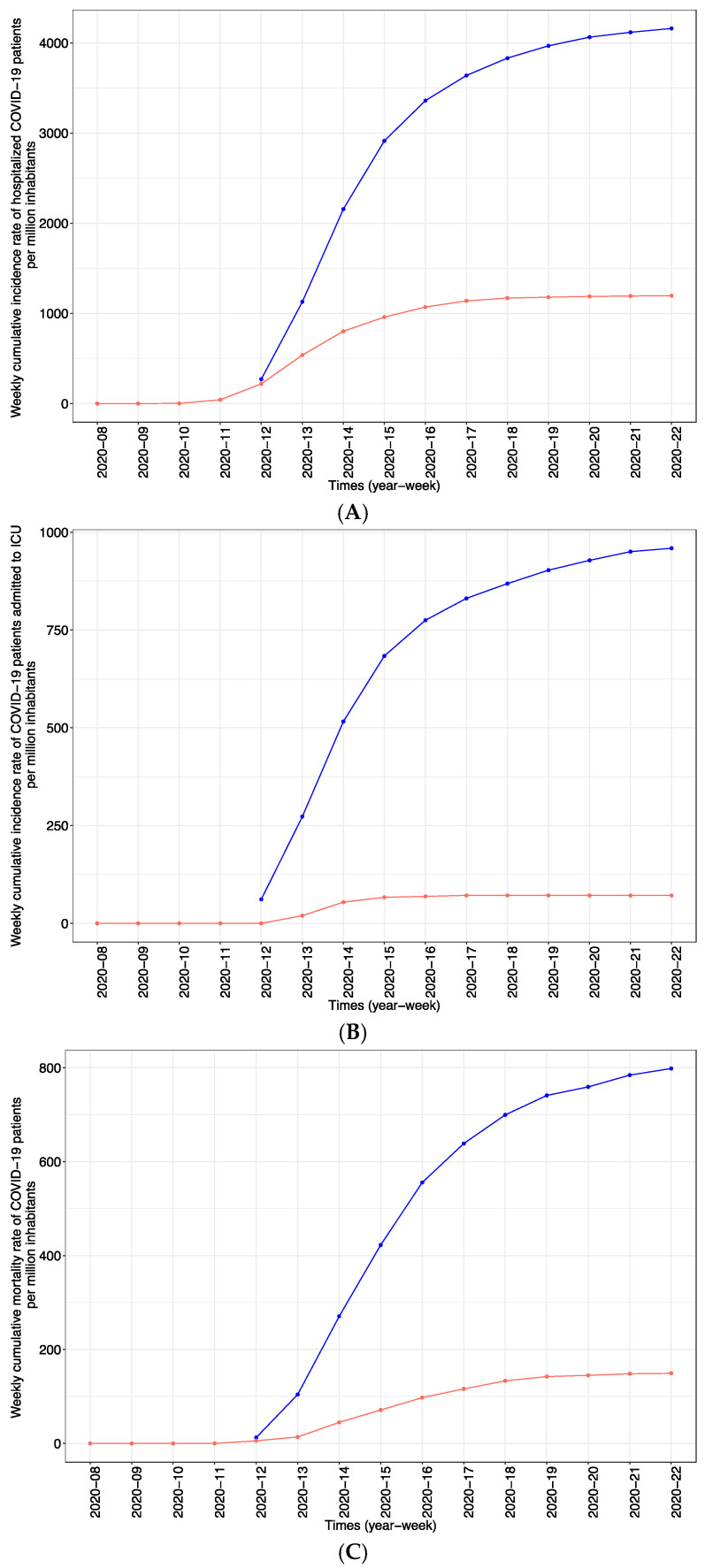
(**A**) Weekly cumulative incidence of hospitalization of COVID-19 patients per million inhabitants in Paris (blue) and Marseille (red). (**B**) Weekly cumulative incidence of intensive care unit (ICU) admission of COVID-19 patients per million inhabitants in Paris (blue) and Marseille (red). (**C**) Weekly cumulative COVID-19-associated mortality per million inhabitants in Paris (blue) and Marseille (red).

Significant differences, particularly in screening and treatment strategies, were observed between Paris, where outpatients were not tested, and Marseille, where outpatients and asymptomatic people were tested. The cumulative incidence rate of hospitalization was 4159 patients per million inhabitants in Paris, which was significantly higher than the rate of 1196 patients per million inhabitants in Marseille (*p*-value < 0.001) (Figure 4A), suggesting a preventive effect associated with broader testing. The number of individuals admitted to the ICU in Paris (959 per million inhabitants) was also significantly higher than that in Marseille (71 per million inhabitants) (*p*-value < 0.001) (Figure 4B); moreover, 798 and 149 COVID-19 deaths per million inhabitants were observed in Paris and Marseille, respectively (Figure 4C). Four patients (3.1%) (one woman and three men) living in Marseille died under the age of 60, including one patient aged 56 years, two patients aged 58 years and one patient aged 59 years. These patients had comorbidities: bronchopulmonary large cell lung cancer metastasizing to the brain, diabetes, hypertension, early Alzheimer’s disease or a history of stroke or severe ischemic heart disease. There was a large amount of missing data on the age of deceased patients in Paris, making analysis of the latter impossible. Paris had an excess mortality of approximately 2000 patients between 2020 and 2018/2019 (+21.2% vs. 2018 and +21.9% vs. 2019), whereas 384 (+7.7%) additional patients died compared to 2018 and 371 (+7.4%) compared to 2019 in Marseille (Table 4). Moreover, there was a drop between −23.8% and 1.7% in mortality in hospice or residential facilities for dependent elderly people in Marseille, which was not the case in Paris (+70.8% vs. 2018 and +61.6% vs. 2019).

## 4. Discussion

The impact of COVID-19 has been very heterogeneous in different countries in the world, but also within Europe. Western European countries as well as the USA have been particularly affected by this emerging disease and have the highest mortality rates, despite high health expenditures and GDP [10]. France has a high overall mortality including in patients under-60 years and a CFR reaching 20% at the time of study period (first period of the pandemic).

Among the measures that have been proposed to achieve better control and lower mortality in different countries, different strategies have been adopted. The global lockdown or restricted confinement of meeting places has not been able to prove its effectiveness. Three countries, namely, Spain, Italy and France, have conducted seroprevalence surveys and showed that locked-down people tended to have more antibodies against SARS-CoV-2 than others, suggesting that they were more prone to be exposed to the virus [20,27,28]. Therefore, the lockdown does not appear to be essential [29,30].

The organization of care also seemed to play an important role. Indeed, saturation of hospital services can lead to the inability to manage admissions at the peak of the epidemic due to a lack of beds or a lack of personnel [29,31]. The lack of beds cannot therefore explain this high mortality, although France is a country where the number of hospital beds is higher than the average for OECD countries [32]. The earliness and magnitude of PCR testing campaigns seemed to be a determining factor both in Europe and in countries in the Far East such as Korea, Taiwan or China [10]. In France, the French government explicitly indicated that screening during the epidemic phase was not necessary [33]. Patients with COVID-19 were instructed to consult emergency services only in case of respiratory difficulties [34]. In his commentary, Minni et al. recalled the importance of early detection for the rapid and effective management of patients [35].

Finally, therapeutic strategies are the subject of complex debates [36]. The use of corticosteroid therapy in seriously ill patients considered as basic care by most encyclopedias but which has been strangely re-tested in the “recovery” trial is probably advisable at relatively low doses. On the other hand, corticosteroid therapy in patients with moderate to medium clinical presentations is not effective and probably leads to an increase in mortality, as was suspected and recently confirmed by the “recovery” study [37]. Finally, remdesivir does not improve clinical status and only slightly shortens the length of hospital stay [37,38,39]. Hydroxychloroquine has shown in a study in 39 public hospitals on 4642 patients in Ile-de-France a shortening of the duration of hospitalization [40]. Finally, a meta-analysis of randomized studies showed a 4.5-day reduction in the duration of symptoms for hydroxychloroquine compared to remdesivir and lopinavir [36]. Thus, the strategy proposed in Marseille with the prescription of hydroxychloroquine and azithromycin may have played a role in the very low mortality observed in a series of 3700 cases [41]. In France, during the first wave of the new virus, only one treatment was officially recommended to reduce fever in COVID-19 cases: paracetamol [34].

Mortality by age group can be examined in comparable situations and mortality in people under 60 years of age can serve as a marker of the effectiveness of therapeutic management in a given situation. In Figure 1, the natural mortality, observable from three ships with outbreaks of CoV-2-SARS on board, was extremely low, suggesting that in other contexts, mortality should be extremely low in healthy people under 50 years of age [12,13,14]. Focusing only on mortality in the under-60 age group, we observed that mortality was lower in Spain and Germany than in France. It was extremely low in patients seen at Marseille IHU and significantly lower still in those who received hydroxychloroquine and azithromycin treatment in all age groups [41]. This also translates into a decrease in all-cause mortality in the Southern region of France compared to France overall. Particularly, in the Ile-de-France region, the mortality rate among people under 60 years of age was twice that in the Southern region. Finally, if we project the estimated number of deaths obtained by multiplying lethality by seroprevalence, the model very clearly shows excess mortality in the Ile-de-France and Grand-Est regions compared to the Bouches-du-Rhône department [31,42]. Overall, it is difficult to dispute that despite similar epidemic levels, there were more hospitalized patients, more transfers to intensive care units and more deaths in Paris than in Marseille, and the correlation of all these parameters suggests that this is not due to chance.

A high genetic diversity of SARS-CoV-2 strains was observed in Marseille during the end of the first wave and during the summer [43]. The spread of a new variant in September in Marseille (named marseille-4, which was found to originate from a mink farmer) [44], supported by the appearance of new variants in the world (UK variant, South African variant, Brazilian variant, etc.) [45,46] may potentially be an explanation for these differences in mortality associated with COVID-19 [47]. Genetic analysis of these first wave strains must be conducted.

Nevertheless, the comparison of mortalities between countries is subject to biases, inherent to the very diverse epidemiological situation according to countries, but also within the same country, as demonstrated by our results. These differences in mortality may also be due to disparities in the populations of each country but also within the same country, notably in terms of co-morbidities, risk factors or epidemiological situation. It is therefore necessary to remember that such descriptive analyses must be adjusted to the local situation at a given time. Unfortunately, comorbidity data are not available in every country including in Paris. In Marseille, a recent study by our team resulted in the calculation of the Charlson comorbidity index to determine the probability of death in the year for patients at the Assistance Publique-Hôpitaux de Marseille in the absence of COVID-19. Over the study period (March to June 2020), 88.8% (out of 178 patients who died) of them had an 85% risk of dying within a year given their comorbidities [48]. These initial results suggest that mortality due to COVID-19 results in a limited number of years of life lost due to advanced age and pre-existing comorbidities in the most vulnerable patients.

## 5. Conclusions

Countries in Europe unquestionably need to think about on the management of their COVID-19 epidemics. It appears that the richest countries with the highest level of care have had significantly higher mortality rates than the poorest countries [29,41]. Among the possible reasons for this difference are the inability to rapidly develop diagnostic tests, including in France during the first months of the epidemic; the lack of immediate medical care for patients [34,38], which was the consequence of the inability to meet the needs in terms of testing whereas China prescribed antivirals [38]; and the inadequacy of influenza-based recommendations as COVID-19 is very different from influenza (e.g., hypercoagulation problems and happy hypoxia), which led to delays in the care and medicalization of these patients [3,7,9,49,50].

Furthermore, we believe that in developed countries, which are less familiar with infectious diseases [29], strategies tending to privilege therapeutic trials over routine care have led to delayed management and less efficient quality of care than in countries where the immediate health of patients was prioritized over therapeutic trials. For example, in China, the idea of not giving specific care was considered unethical, and doctors prescribed antivirals, but no more than three [38].

Regarding France, the strategy was to give no treatment until its effectiveness had been proven by randomized trials. This difference in strategies between the rich Western world and the rest of the world is probably relevant to the very high mortality rate observed in France.

## Figures and Tables

**Table 1:** Comparison of government policies against COVID-19.

Countries	Number of COVID-19 Tests per 1000 Inhabitants	Lockdown	Overall Government Response Severity Index	Date Where the Severity Index Is Max
Italy	64.7	Require not leaving house with minimal exceptions (e.g., allowed to leave once a week, or only one person can leave at a time, etc.)	93.52	12 April 2020
France	13	Require not leaving house with exceptions for daily exercise, grocery shopping, and “essential” trips	90.74	17 March 2020
Spain	54.2	Require not leaving house with exceptions for daily exercise, grocery shopping, and “essential” trips	85.19	30 March 2020
Belgium	60.2	Require not leaving house with exceptions for daily exercise, grocery shopping, and “essential” trips	81.48	20 March 2020
Netherlands	20.4	Require not leaving house with exceptions for daily exercise, grocery shopping, and “essential” trips	79.63	31 March 2020
Norway	45.3	No measures	75.93	24 March 2020
United Kingdom	31.6	Require not leaving house with exceptions for daily exercise, grocery shopping, and “essential” trips	75.93	26 March 2020
Germany	47.2	Require not leaving house with exceptions for daily exercise, grocery shopping, and “essential” trips	73.15	22 March 2020
Denmark	91.3	Recommend not leaving house	72.22	18 March 2020
Finland	33.4	Recommend not leaving house	68.52	28 March 2020
Iceland	179.0	No measures	53.7	20 March 2020
Sweden	23.7	No measures	46.3	24 April 2020

**Table 2:** Excess mortality in 2020 compared to 2018/2019 from January to August in France, including 4 regions. The data were collected from the INSEE database.

	**2020 vs. 2018**									
	**Sud**		**Ile-de-France**		**Grand-Est**		**New-Aquitaine**		**France**	
	***n***	**Number of Excess Deaths (% Excess)**	***n***	**Number of Excess Deaths (% Excess)**	***n***	**Number of Excess Deaths (% Excess)**	***n***	**Number of Excess Deaths (% Excess)**	***n***	**Number of Excess Deaths (% Excess)**
Deaths from all causes	34,176	321 (0.9%)	50,386	10,966 (21.8%)	36,194	4150 (11.5%)	44,017	−1175 (−2.7%)	411,271	18,983 (4.6%)
Deaths from all causes in public or private hospitals	17,695	−1039 (−5.9%)	30,790	4622 (15.0%)	21,099	1017 (4.8%)	22,485	−1063 (−4.7%)	219,669	−1104 (−0.5%)
Deaths from all causes in hospice or among dependent elderly residents in retirement homes	3981	190 (4.8%)	5196	2493 (48.0%)	5078	1463 (28.8%)	6859	−3 (0.0%)	51,634	5908 (11.4%)
Deaths from all causes at home	9721	597 (6.1%)	10,674	3119 (29.2%)	7979	1083 (13.6%)	10,931	100 (0.9%)	98,624	7060 (7.2%)
	**2020 vs. 2019**									
	**Sud**		**Ile-de-France**		**Grand-Est**		**New-Aquitaine**		**France**	
	***n***	**Number of Excess Deaths (% Excess)**	***n***	**Number of Excess Deaths (% Excess)**	***n***	**Number of Excess Deaths (% Excess)**	***n***	**Number of Excess Deaths (% Excess)**	***n***	**Number of Excess Deaths (% Excess)**
Deaths from all causes	34,267	230 (0.7%)	50,556	10,796 (21.4%)	35,654	4690 (13.2%)	43,791	−949 (−2.2%)	409,835	20,419 (5.0%)
Deaths from all causes in public or private hospital	17,784	−1128 (−6.3%)	31,045	4367 (14.1%)	20,534	1582 (7.7%)	22,266	−844 (−3.8%)	217,409	1156 (0.5%)
Deaths from all causes in hospice or among dependent elderly residents in retirement homes	4135	36 (0.9%)	4952	2737 (55.3%)	4862	1679 (34.5%)	6473	383 (5.9%)	49,578	7964 (16.1%)
Deaths from all causes at home	9659	659 (6.8%)	10,843	2950 (27.2%)	7976	1086 (13.6%)	10,686	345 (3.2%)	96,429	9255 (9.6%)

**Table 3:** Mortality estimation according to the prevalence of antibodies after a delay.

Region	Population Size (Inhabitants)	Seroprevalence (%)	Number of Estimated COVID-19 Cases on the Basis of Seroprevalence	Number of Estimated Deaths Using a 0.5% Probability of Death	Estimated COVID-19 Mortality per Million Inhabitants	Number of Observed Deaths (as of 2 June)	Ratio of Estimated to Observed Deaths	COVID-19 Mortality per Million Inhabitants
Ile-de-France	12,278,210	10	1,227,821	6139	500	7273	0.84	592
Grand-Est	5,511,747	9	496,057	2480	450	3565	0.70	647
New-Aquitaine	5,999,982	3.1	185,999	930	155	420	2.21	70
Bouches-du-Rhône	2,034,469	7.96	161,944	810	398	535	1.51	263

**Table 4:** Excess mortality in 2020 compared to 2018/2019 from January to August in Marseille and Paris. The data were collected from the INSEE database.

	2020 vs. 2018				2020 vs. 2019			
	Paris		Marseille		Paris		Marseille	
	*n*	Number of Excess Deaths (% Excess)	*n*	Number of Excess Deaths (% excess)	*n*	Number of Excess Deaths (% Excess)	*n*	Number of Excess Deaths (% Excess)
Deaths from all causes	9243	1961 (21.2%)	5015	384 (7.7%)	9192	2012 (21.9%)	5028	371 (7.4%)
Deaths from all causes in public or private hospitals	5960	806 (13.5%)	2852	−164 (−5.8%)	5830	936 (16.1%)	2761	−73 (−2.6%)
Deaths from all causes in hospice or among dependent elderly residents in retirement homes	565	400 (70.8%)	248	59 (23.8%)	597	368 (61.6%)	302	5 (1.7%)
Deaths from all causes at home	2319	737 (31.8)	1732	70 (4.0%)	2426	630 (26.0%)	1812	−10 (0.6%)

## Data Availability

International or French data are available through the web links provided in the materials and methods. The Marseille data are not available in the public domain, but anyone interested in using the data for scientific purpose is free to request permission from the corresponding author: Didier Raoult (didier.raoult@gamil.com).

## Supplementary Materials

The following are available online at https://www.mdpi.com/article/10.3390/jcm10132942/s1, Supplementary Data S1: Deaths from all causes among people living in the 4 regions in France. Supplementary Data S2: Deaths from all causes in public or private hospitals among people living in the 4 regions in France. Supplementary Data S3: Deaths from all causes in hospice or among dependent elderly residents in retirement homes in the 4 regions in France. Supplementary Data S4: Deaths from all causes at home in the 4 regions in France. Supplementary Table S1: Age classes of COVID-19-associated fatalities at IHU Méditerranée Infection, at AP-HM, in French regions and in France overall. Supplementary Table S2: COVID-19-associated fatalities in patients under 60 years of age.

## References

[1] Dong E., Du H., Gardner L. (2020). An interactive web-based dashboard to track, COVID-19 in real time. Lancet Infect. Dis..

[2] Vanhems P. (2020). SARS-CoV2 infection and primary school closure. Eurosurveillance.

[3] Moatti J.P. (2020). The French response to COVID-19: Intrinsic difficulties at the interface of science, public health, and policy. Lancet Public Health.

[4] Décret n 2020-260 du 16 Mars 2020 Portant Réglementation des Déplacements dans le Cadre de la Lutte Contre la Propagation du Virus COVID-19. https://www.legifrance.gouv.fr/affichTexte.do?cidTexte=JORFTEXT000041728476.

[5] Gudbjartsson D.F., Helgason A., Jonsson H., Magnusson O.T., Melsted P., Norddahl G.L., Saemundsdottir J., Sigurdsson A., Sulem P., Agustsdottir A.B. (2020). Spread of SARS-CoV-2 in the Icelandic Population. N. Engl. J. Med..

[6] Kwon K.T., Ko J.H., Shin H., Sung M., Kim J.Y. (2020). Drive-Through Screening Center for COVID-19: A Safe and Efficient Screening System against Massive Community Outbreak. J. Korean Med. Sci..

[7] Définition de cas d’infection au SARS-CoV-2 (COVID-19) Mise à jour le 13 Mars 2020 (n.d.). http://splf.fr/wp-content/uploads/2020/03/COVID-19_definition_cas_20200313.pdf.

[8] Gautret P., Lagier J.C., Parola P., Meddeb L., Mailhe M., Doudier B., Courjon J., Giordanengo V., Vieira V.E., Dupont H.T. (2020). Hydroxychloroquine and azithromycin as a treatment of COVID-19: Results of an open-label non-randomized clinical trial. Int. J. Antimicrob. Agents.

[9] Décret n 2020-314 du 25 Mars 2020 Prescrivant les Mesures Générales Nécessaires Pour Faire Face à l’épidémie COVID-19 dans le Cadre de L’état D’urgence Sanitaire. https://www.legifrance.gouv.fr/affichTexte.do?cidTexte=JORFTEXT000041755775&categorieLien=id.

[10] Our World in Data Daily COVID-19 Tests per Thousand People. https://ourworldindata.org/coronavirus-testing.

[11] Coronavirus Government Response Tracker | Blavatnik School of Government. https://www.bsg.ox.ac.uk/research/research-projects/coronavirus-government-response-tracker#data.

[12] Russell T.W., Hellewell J., Jarvis C.I., Van Zandvoort K., Abbott S., Ratnayake R., Flasche S., Eggo R.M., Edmunds W.J., Kucharski A.J. (2020). Estimating the infection and case fatality ratio for coronavirus disease (COVID-19) using age-adjusted data from the outbreak on the Diamond Princess cruise ship February 2020. Eurosurveillance.

[13] Alvarado G.R., Pierson B.C., Teemer E.S., Gama H.J., Cole R.D., Jang S.S. (2020). Symptom Characterization and Outcomes of Sailors in Isolation After a COVID-19 Outbreak on a US Aircraft Carrier. JAMA Netw. Open.

[14] Communiqué_Publication des Conclusions des Enquêtes sur la Contamination au COVID-19 au sein du Groupe Aéronaval. https://www.defense.gouv.fr/salle-de-presse/communiques/communique_publication-des-conclusions-des-enquetes-sur-la-contamination-au-covid-19-au-sein-du-groupe-aeronaval.

[15] Téléchargement des Fichiers des décès Quotidiens−Nombre de décès Quotidiens|Insee. https://www.insee.fr/fr/statistiques/4487988?sommaire=4487854.

[16] What Is a Z-Score?—EUROMOMO. https://www.euromomo.eu/how-it-works/what-is-a-z-score/.

[17] Données Hospitalières Relatives à L’épidémie de COVID-19-Data.Gouv.fr. https://www.data.gouv.fr/fr/datasets/donnees-hospitalieres-relatives-a-lepidemie-de-covid-19/#_.

[18] Population par Région et Département-Structure de la Population-France-Les Chiffres-Ined-Institut National D’études Démographiques. https://www.ined.fr/fr/tout-savoir-population/chiffres/france/structure-population/regions-departements/.

[19] Géodes-Santé Publique France. https://geodes.santepubliquefrance.fr/#c=home.

[20] Carrat F., De Lamballerie X., Rahib D., Blanché H., Lapidus N., Artaud F., Kab S., Renuy A., de Edelenyi F.S., Meyer L. (2020). Seroprevalence of SARS-CoV-2 among adults in three regions of France following the lockdown and associated risk factors: A multicohort study. MedRxiv.

[21] Salje H., Tran Kiem C., Lefrancq N., Salje H., Kiem C.T., Lefrancq N., Courtejoie N., Bosetti P., Paireau J., Andronico A. (2020). Estimating the burden of SARS-CoV-2 in France. Science.

[22] Abat C., Chaudet H., Colson P., Rolain J.M., Raoult D. (2015). Real-Time Microbiology Laboratory Surveillance System to Detect Abnormal Events and Emerging Infections Marseille France. Emerg. Infect. Dis..

[23] Colson P., Rolain J.M., Abat C., Charrel R., Fournier P.E., Raoult D. (2015). EPIMIC: A Simple Homemade Computer Program for Real-Time EPIdemiological Surveillance and Alert Based on MICrobiological Data. PLoS ONE.

[24] Recensement de la Population| Insee. https://webcache.googleusercontent.com/search?q=cache:7m-ySsbBFKYJ:https://www.insee.fr/fr/statistiques/fichier/4265439/dep13.pdf+&cd=2&hl=fr&ct=clnk&gl=fr.

[25] R Core Team R: A Language and Environment for Statistical Computing. R Foundation for Statistical Computing Vienna Austria. http://www.R-project.org/.

[26] Wickham H. (2016). ggplot2: Elegant Graphics for Data Analysis.

[27] Pollán M., Pérez-Gómez B., Pastor-Barriuso R., Oteo J., Hernán M.A., Sanmartín J.L., Fernández-García A., Cruz I., de Larrea N.F., Molina M. (2020). Prevalence of SARS-CoV-2 in Spain (ENE-COVID): A nationwide, population-based seroepidemiological study. Lancet.

[28] Istituto Nazionale di Statistica Ministera della Salute Primi Risultati Dell’indagine di Sieroprevalenza sul SARS-COV2, Comunicato stampa. https://www.istat.it/it/files//2020/08/ReportPrimiRisultatiIndagineSiero.pdf.

[29] De Larochelambert Q., Marc A., Antero J., Le Bourg E., Toussaint J.F. (2020). Covid-19 Mortality: A Matter of Vulnerability Among Nations Facing Limited Margins of Adaptation. Front. Public Health.

[30] Bendavid E., Oh C., Bhattacharya J., Ioannidis J.P.A. (2021). Assessing mandatory stay-at-home and business closure effects on the spread of COVID-19. Eur. J. Clin. Investig..

[31] Souris M., Gonzalez J.P. (2020). COVID-19: Spatial analysis of hospital case-fatality rate in France. PLoS ONE.

[32] OECD (2019). Health at a Glance 2019: OECD Indicators.

[33] Préparation a la phase épidémique de COVID-19 Ministère des Solidarités et de la Santé 16 March 2020. https://solidaritessante.gouv.fr/IMG/pdf/guide-covid-19-phaseepidemique-v15-16032020.pdf.

[34] Why Should I Isolate Myself? Until When Should I Isolate Myself?. https://solidarites-sante.gouv.fr.

[35] Minni A., Ralli M., Candelori F., Cialente F., Ercoli L., Parlapiano C., Greco A., De Vincentiis M. (2021). Lessons learned from COVID-19 pandemic in Italy—A commentary. Bosn. J. Basic Med. Sci..

[36] Siemieniuk R.A., Bartoszko J.J., Ge L., Zeraatkar D., Izcovich A., Kum E., Pardo-Hernandez H., Rochwerg B., Lamontagne F., Han M.A. (2020). Drug treatments for covid-19: Living systematic review and network meta-analysis. BMJ.

[37] Horby P., Lim W.S., Emberson J.R., Mafham M., Bell J.L., Linsell L., Staplin N., Brightling C., Ustianowski A., Elmahi E. (2020). Dexamethasone in Hospitalized Patients with Covid-19—Preliminary Report. N. Engl. J. Med..

[38] Wang Y., Zhang D., Du G., Du R., Zhao J., Jin Y., Fu S., Gao L., Cheng Z., Lu Q. (2020). Remdesivir in adults with severe COVID-19: A randomised, double-blind, placebo-controlled, multicentre trial. Lancet.

[39] Young B., Tan T.T., Leo Y.S. (2021). The place for remdesivir in COVID-19 treatment. Lancet Infect. Dis..

[40] Sbidian E., Josse J., Lemaitre G., Mayer I., Bernaux M., Gramfort A., Lapidus N., Paris N., Neuraz A., Lerner I. (2020). Hydroxychloroquine with or without azithromycin and in-hospital mortality or discharge in patients hospitalized for COVID-19 infection: A cohort study of 4642 in-patients in France. MedRxiv.

[41] Lagier J.C., Million M., Gautret P., Colson P., Cortaredona S., Giraud-Gatineau A., Honoré S., Gaubert J.Y., Fournier P.E., Tissot-Dupont H. (2020). Outcomes of 3,737 COVID-19 patients treated with hydroxychloroquine/azithromycin and other regimens in Marseille France: A retrospective analysis. Travel Med. Infect. Dis..

[42] Courtejoie N., Dubost C.L. (2020). Parcours Hospitaliers des Patients Atteints de la Covid-19 lors de la Première Vague de l’épidémie. Les Dossiers de la. DRESS N°67. https://drees.solidarites-sante.gouv.fr/sites/default/files/2020-10/DD67.pdf.

[43] Colson P., Levasseur A., Gautret P., Fenollar F., Thuan Hoang V., Delerce J., Bitam I., Saile R., Maaloum M., Padane A. (2021). Introduction into the Marseille geographical area of a mild SARS-CoV-2 variant originating from sub-Saharan Africa: An investigational study. Travel Med. Infect. Dis..

[44] Fournier P.E., Colson P., Levasseur A., Devaux C.A., Gautret P., Bedotto M., Delerce J., Brechard L., Pinault L., Lagier J.C. (2021). Emergence and outcomes of the SARS-CoV-2 ‘Marseille-4’ variant. Int. J. Infect. Dis..

[45] Wise J. (2020). Covid-19: New coronavirus variant is identified in UK. BMJ.

[46] Makoni M. (2021). South Africa responds to new SARS-CoV-2 variant. Lancet.

[47] Jain A., Rophina M., Mahajan S., Krishnan B.B., Sharma M., Mandal S., Fernandez T., Sultanji S., Jolly B., Mathew S. (2021). Analysis of the potential impact of genomic variants in global SARS-CoV-2 genomes on molecular diagnostic assays. Int. J. Infect. Dis..

[48] Arlotto S., Gares A., Giraud-Gatineau A., Lagier J.-C., Jimeno M.T., Peretti-Watel P., Million M., Parola P., Brouqui P., Raoult D. Life-Years Lost by COVID-19 Patients in Public Hospitals of Marseille (APHM- South-Eastern France): A Limited Death Toll. https://www.mediterranee-infection.com/wp-content/uploads/2020/04/2021-01-26bis-DEATHCOVID.pdf.

[49] Brouqui P., Amrane S., Million M., Cortaredona S., Parola P., Lagier J.C., Raoult D. (2020). Asymptomatic hypoxia in COVID-19 is associated with poor outcome. Int. J. Infect. Dis..

[50] Camoin-Jo L., Gautret P., Colson P., Lagier J.C., Tissot-Dupont H., Million M., Giraud-Gatineau A., Boudjema S., Chaudet H., Raoult D. High Prevalence of Lupus Anticoagulant in Ambulatory COVID-19 Patients: Interest of Hydroxychloroquine?-IHU. https://www.mediterranee-infection.com/high-prevalence-of-lupus-anticoagulant-in-ambulatory-covid-19-patients-interest-of-hydroxychloroquine/.

