# Supplementary material for: Evaluation of Strategies to Fight COVID-19: The French Paradigm
Source: J Clin Med. 2021 Jun 30;10(13):2942. doi: 10.3390/jcm10132942 (PMC8268313; doi:10.3390/jcm10132942)
Supplement: Supplementary file 1 [file jcm-10-02942-s001.zip › jcm-1229028-supplementary.pdf]

Supplementary data

Title: Evaluation of strategies to fight COVID-19: The French paradigm

Author list:

Audrey GIRAUD-GATINEAU[1,2,3,4] (PhD student); Philippe GAUTRET[1,2] (MD, PhD); Philippe COLSON[1,5]; Hervé CHAUDET[1,2,3] (MD); Didier RAOULT[1,5*] (MD, PhD)

Affiliations:

[1] IHU Méditerranée Infection, 19-21 boulevard Jean Moulin, 13005 Marseille, France;

[2] Aix Marseille Univ., Institut de Recherche pour le Développement (IRD), Assistance Publique – Hôpitaux de Marseille (AP-HM), Service de Santé des Armées (SSA), Vecteurs – Infections Tropicales et Méditerranéennes (VITROME), Marseille, France;

[3] French Armed Forces Center for Epidemiology and Public Health (CESPA), Service de Santé des Armées (SSA), Marseille, France;

[4] Assistance Publique- Hôpitaux de Marseille (AP-HM), Marseille, France;

[5] Aix-Marseille Univ., Institut de Recherche pour le Développement (IRD), Assistance Publique - Hôpitaux de Marseille (AP-HM), Microbes Evolution Phylogeny and Infections (MEPHI), 27 boulevard Jean Moulin, 13005 Marseille, France;

* Corresponding author: Didier Raoult, IHU Méditerranée Infection, 19-21 boulevard Jean Moulin, 13005 Marseille, France. Tel.: +33 413 732 401; Fax: +33 413 732 402; email: didier.raoult@gmail.com

Key words: COVID-19; SARS-CoV-2; France; deaths; Marseille; Paris

Supplementary data S1

Deaths from all causes among people living in the Sud region	2018	2019	2020
**January**	5207	5137	4781
**% excess**	-8,2	-6,9	
**February**	4361	4732	4256
**% excess**	-2,4	-10,1	
**March**	4707	4480	4852
**% excess**	3,1	8,3	
**April**	4040	4047	4725
**% excess**	17,0	16,8	
**May**	3945	3977	3956
**% excess**	0,3	-0,5	
**June**	3767	3846	3893
**% excess**	3,3	1,2	
**July**	3958	4060	3982
**% excess**	0,6	-1,9	
**August**	4191	3988	4052
**% excess**	-3,3	1,6	
**TOTAL Jan-Aug**	34176	34267	34497
**Excess deaths/2020**	321	230	
**% excess**	0,9	0,7	

Deaths from all causes among people living in Ile-de-France	2018	2019	2020
**January**	7366	7642	7163
**% excess**	-2,8	-6,3	
**February**	6442	6879	6277
**% excess**	-2,6	-8,8	
**March**	7291	6388	9746
**% excess**	33,7	52,6	
**April**	6117	6030	14221
**% excess**	132,5	135,8	
**May**	6032	6008	6569
**% excess**	8,9	9,3	
**June**	5584	5826	5747
**% excess**	2,9	-1,4	
**July**	6052	6051	5629
**% excess**	-7,0	-7,0	
**August**	5502	5732	6000
**% excess**	9,1	4,7	
**TOTAL Jan-Aug**	50386	50556	61352
**Excess deaths/2020**	10966	10796	
**% excess**	21,8	21,4	

Deaths from all causes among people living in Grand-Est	2018	2019	2020
**January**	5164	5165	4986
**% excess**	-3,4	-3,5	
**February**	4608	4846	4583
**% excess**	-0,5	-5,4	
**March**	5641	4772	7057
**% excess**	25,1	47,9	
**April**	4445	4288	7081
**% excess**	59,3	65,1	
**May**	4078	4269	4282
**% excess**	5,0	0,3	
**June**	3818	4030	4033
**% excess**	5,6	0,1	
**July**	4346	4182	4028
**% excess**	-7,3	-3,7	
**August**	4094	4102	4294
**% excess**	4,9	4,7	
**TOTAL Jan-Aug**	36194	35654	40344
**Excess deaths/2020**	4150	4690	
**% excess**	11,5	13,2	

Deaths from all causes among people living in New-Aquitaine	2018	2019	2020
**January**	6188	6552	6115
**% excess**	-1,2	-6,7	
**February**	5726	5947	5492
**% excess**	-4,1	-7,7	
**March**	6463	5703	5978
**% excess**	-7,5	4,8	
**April**	5442	5271	5304
**% excess**	-2,5	0,6	
**May**	5128	5321	5018
**% excess**	-2,1	-5,7	
**June**	4964	4970	4864
**% excess**	-2,0	-2,1	
**July**	5120	5085	5100
**% excess**	-0,4	0,3	
**August**	4986	4942	4971
**% excess**	-0,3	0,6	
**TOTAL Jan-Aug**	44017	43791	42842
**Excess deaths/2020**	-1175	-949	
**% excess**	-2,7	-2,2	

Supplementary data S2

Deaths from all causes in public or private hospitals among people living in the Sud region	**2 018**	**2 019**	**2 020**
**January**	2 679	2 666	2 486
**% excess**	-7	-7	
**February**	2 203	2 420	2 197
**% excess**	0	-9	
**March**	2 392	2 327	2 080
**% excess**	-13	-11	
**April**	2 078	2 156	2 070
**% excess**	0	-4	
**May**	2 106	2 032	1 865
**% excess**	-11	-8	
**June**	2 009	2 029	1 924
**% excess**	-4	-5	
**July**	2 079	2 106	1 995
**% excess**	-4	-5	
**August**	2 149	2 048	2 039
**% excess**	-5	0	
**TOTAL Jan-Aug**	17 695	17 784	16 656
**Excess deaths/2020**	-1 039	-1 128	
**% excess**	-5,9	-6,3	

Deaths from all causes in public or private hospitals among people living in Ile-de-France	**2 018**	**2 019**	**2 020**
**January**	4 421	4 642	4 344
**% excess**	-2	-6	
**February**	3 923	4 135	3 859
**% excess**	-2	-7	
**March**	4 356	3 899	5 624
**% excess**	29	44	
**April**	3 701	3 766	7 388
**% excess**	100	96	
**May**	3 757	3 621	3 787
**% excess**	1	5	
**June**	3 518	3 643	3 418
**% excess**	-3	-6	
**July**	3 744	3 750	3 397
**% excess**	-9	-9	
**August**	3 370	3 589	3 595
**% excess**	7	0	
**TOTAL Jan-Aug**	30 790	31 045	35 412
**Excess deaths/2020**	4 622	4 367	
**% excess**	15,0	14,1	

Deaths from all causes in public or private hospitals among people living in Grand-Est	**2 018**	**2 019**	**2 020**
**January**	3 016	2 899	2 822
**% excess**	-6	-3	
**February**	2 685	2 799	2 580
**% excess**	-4	-8	
**March**	3 195	2 741	3 928
**% excess**	23	43	
**April**	2 538	2 459	3 632
**% excess**	43	48	
**May**	2 363	2 464	2 269
**% excess**	-4	-8	
**June**	2 298	2 368	2 235
**% excess**	-3	-6	
**July**	2 580	2 427	2 208
**% excess**	-14	-9	
**August**	2 424	2 377	2 442
**% excess**	1	3	
**TOTAL Jan-Sep 7**	21 099	20 534	22 116
**Excess deaths/2020**	1 017	1 582	

Deaths from all causes in public or private hospitals among people living in New-Aquitaine	**2 018**	**2 019**	**2 020**
**January**	3 169	3 277	3 141
**% excess**	-1	-4	
**February**	2 942	2 946	2 823
**% excess**	-4	-4	
**March**	3 253	2 855	2 944
**% excess**	-9	3	
**April**	2 749	2 643	2 461
**% excess**	-10	-7	
**May**	2 660	2 810	2 451
**% excess**	-8	-13	
**June**	2 532	2 650	2 469
**% excess**	-2	-7	
**July**	2 641	2 608	2 545
**% excess**	-4	-2	
**August**	2 539	2 477	2 588
**% excess**	2	4	
**TOTAL Jan-Aug**	22 485	22 266	21 422
**Excess deaths/2020**	-1 063	-844	

% excess	4,8	7,7		% excess	-4,7	-3,8

Supplementary data S3

Deaths from all causes in hospice or among dependent elderly residents in retirement homes in th Sud region	2018	2019	2020
January	630	587	593
% excess	-5,9	1,0	
February	513	621	468
% excess	-8,8	-24,6	
March	605	520	612
% excess	1,2	17,7	
April	466	464	701
% excess	50,4	51,1	
May	443	507	433
% excess	-2,3	-14,6	
June	397	469	456
% excess	14,9	-2,8	
July	448	458	453
% excess	1,1	-1,1	
August	479	509	455
% excess	-5,0	-10,6	
TOTAL Jan-Aug	3981	4135	4171
Excess deaths/2020	190	36	
% excess	4,8	0,9	

Deaths from all causes in hospice or among dependent elderly residents in retirement homes in Ile-de-France	2018	2019	2020
January	786	771	693
% excess	-11,8	-10,1	
February	680	711	625
% excess	-8,1	-12,1	
March	860	635	1210
% excess	40,7	90,6	
April	645	573	2695
% excess	317,8	370,3	
May	597	625	728
% excess	21,9	16,5	
June	517	539	589
% excess	13,9	9,3	
July	576	551	553
% excess	-4,0	0,4	
August	535	547	596
% excess	11,4	9,0	
TOTAL Jan-Aug	5196	4952	7689
Excess deaths/2020	2493	2737	
% excess	48,0	55,3	

Deaths from all causes in hospice or among dependent elderly residents in retirement homes in Grand-Est	2018	2019	2020
January	679	718	695
% excess	2,4	-3,2	
February	640	680	671
% excess	4,8	-1,3	
March	896	693	1144
% excess	27,7	65,1	
April	659	587	1466
% excess	122,5	149,7	
May	597	539	687
% excess	15,1	27,5	
June	486	500	605
% excess	24,5	21,0	
July	562	582	632
% excess	12,5	8,6	
August	559	563	641
% excess	14,7	13,9	
TOTAL Jan-Aug	5078	4862	6541
Excess deaths/2020	1463	1679	

Deaths from all causes in hospice or among dependent elderly residents in retirement homes in New-Aquitaine	2018	2019	2020
January	989	1010	961
% excess	-2,8	-4,9	
February	889	935	829
% excess	-6,7	-11,3	
March	1056	863	944
% excess	-10,6	9,4	
April	852	813	940
% excess	10,3	15,6	
May	773	747	781
% excess	1,0	4,6	
June	790	653	799
% excess	1,1	22,4	
July	757	700	836
% excess	10,4	19,4	
August	753	752	766
% excess	1,7	1,9	
TOTAL Jan-Aug	6859	6473	6856
Excess deaths/2020	-3	383	

**% excess**	28,8	34,5

**% excess**	0,0	5,9

Supplementary data S4

Deaths from all causes at home among people living in the Sud region	**2018**	**2019**	**2020**
**January**	1500	1459	1320
**% excess**	-12,0	-9,5	
**February**	1245	1345	1206
**% excess**	-3,1	-10,3	
**March**	1313	1291	1348
**% excess**	2,7	4,4	
**April**	1161	1117	1504
**% excess**	29,5	34,6	
**May**	1109	1122	1307
**% excess**	17,9	16,5	
**June**	1075	1066	1209
**% excess**	12,5	13,4	
**July**	1096	1156	1211
**% excess**	10,5	4,8	
**August 24th**	1222	1103	1213
**% excess**	-0,7	10,0	
**TOTAL Jan-Aug**	9721	9659	10318
**Excess deaths/2020**	597	659	
**% excess**	6,1	6,8	

Deaths from all causes at home among people living in Ile-de-France	**2018**	**2019**	**2020**
**January**	1626	1675	1604
**% excess**	-1,4	-4,2	
**February**	1388	1517	1342
**% excess**	-3,3	-11,5	
**March**	1564	1410	2256
**% excess**	44,2	60,0	
**April**	1319	1312	3220
**% excess**	144,1	145,4	
**May**	1233	1341	1554
**% excess**	26,0	15,9	
**June**	1106	1212	1308
**% excess**	18,3	7,9	
**July**	1259	1244	1209
**% excess**	-4,0	-2,8	
**August 24th**	1179	1132	1300
**% excess**	10,3	14,8	
**TOTAL Jan-Aug**	10674	10843	13793
**Excess deaths/2020**	3119	2950	
**% excess**	29,2	27,2	

Deaths from all causes at home among people living in Grand-Est	**2018**	**2019**	**2020**
**January**	1176	1220	1115
**% excess**	-5,2	-8,6	
**February**	1043	1070	1006
**% excess**	-3,5	-6,0	
**March**	1245	1044	1542
**% excess**	23,9	47,7	
**April**	1027	988	1560
**% excess**	51,9	57,9	
**May**	871	950	1016
**% excess**	16,6	6,9	
**June**	795	907	958
**% excess**	20,5	5,6	
**July**	941	893	920
**% excess**	-2,2	3,0	
**August 24th**	881	904	945
**% excess**	7,3	4,5	
**TOTAL Jan-Sep 7**	7979	7976	9062
**Excess of deaths/2020**	1083	1086	
**% excess**	13,6	13,6	

Deaths from all causes at home among people living in New-Aquitaine	**2018**	**2019**	**2020**
**January**	1536	1634	1470
**% excess**	-4,3	-10,0	
**February**	1485	1532	1319
**% excess**	-11,2	-13,9	
**March**	1644	1394	1603
**% excess**	-2,5	15,0	
**April**	1364	1271	1470
**% excess**	7,8	15,7	
**May**	1245	1254	1380
**% excess**	10,8	10,0	
**June**	1228	1180	1229
**% excess**	0,1	4,2	
**July**	1240	1209	1319
**% excess**	6,4	9,1	
**August 24th**	1189	1212	1241
**% excess**	4,4	2,4	
**TOTAL Jan-Sep 7**	10931	10686	11031
**Excess of deaths/2020**	100	345	
**% excess**	0,9	3,2	

Supplementary table S1 – Age classes of COVID-19-associated fatalities at IHU Méditerranée Infection, at AP-HM, in French regions and in France overall.

	**0-9 N (%)**	**9-19 N (%)**	**20-29 N (%)**	**30-39 N (%)**	**40-49 N (%)**	**50-59 N (%)**	**60-69 N (%)**	**70-79 N (%)**	**80-89 N (%)**	**90 and more N (%)**	**TOTAL**
**IHU Méditerranée Infection**	0 (0)	0 (0)	0 (0)	0 (0)	0 (0)	1 (2.3)	2 (4.7)	14 (32.6)	18 (41.9)	8 (18.6)	43
**AP-HM total**	0 (0)	0 (0)	0 (0)	0 (0)	0 (0)	4 (3.1)	12 (9.2)	33 (25.4)	49 (37.7)	32 (24.6)	130
**Auvergne – Rhône-Alpes**	0 (0)	0 (0)	1 (0.1)	4 (0.2)	12 (0.7)	44 (2.6)	130 (7.8)	340 (20.4)	705 (42.4)	428 (25.7)	1,664
**Bourgogne-Franche-Comté**	0 (0)	0 (0)	0 (0)	3 (0.3)	6 (0.6)	25 (2.5)	86 (8.5)	207 (20.5)	448 (44.3)	236 (23.3)	1,011
**Bretagne**	0 (0)	0 (0)	0 (0)	0 (0)	1 (0.4)	8 (3.2)	28 (11.1)	52 (20.6)	93 (36.9)	70 (27.8)	252
**Centre – Val de Loire**	0 (0)	0 (0)	1 (0.2)	2 (0.4)	3 (0.6)	13 (2.6)	37 (7.3)	86 (16.9)	200 (39.4)	166 (32.7)	508
**Corse**	0 (0)	0 (0)	0 (0)	0 (0)	1 (1.7)	1 (1.7)	2 (3.4)	16 (27.7)	25 (43.1)	13 (22.4)	58
**Grand-Est**	1 (0.03)	0 (0)	2 (0.1)	16 (0.5)	35 (1.0)	98 (2.9)	366 (10.7)	851 (24.8)	1,391 (40.6)	669 (19.5)	3,429
**Hauts-de-France**	0 (0)	0 (0)	1 (0.1)	4 (0.2)	12 (0.7)	70 (4.0)	231 (13.3)	378 (21.7)	688 (39.6)	354 (20.4)	1,738
**Ile-de-France**	2 (0.03)	3 (0.04)	12 (0.2)	49 (0.7)	132 (1.9)	503 (7.1)	1,039 (14.7)	1,665 (23.5)	2,272 (32,0)	1,412 (19.9)	7,089
**Normandie**	0 (0)	0 (0)	0 (0)	2 (0.5)	10 (2.4)	16 (3.8)	56 (13.3)	93 (22.1)	163 (38.8)	80 (19.0)	420
**New-Aquitaine**	0 (0)	0 (0)	2 (0.5)	2 (0.5)	2 (0.5)	13 (3.3)	38 (9.5)	90 (22.6)	152 (38.2)	99 (24.9)	398
**Occitanie**	0 (0)	0 (0)	1 (0.1)	1 (0.1)	3 (0.3)	17 (1.9)	58 (6.4)	102 (11.3)	191 (21.1)	124 (13.7)	497
**Sud**	0 (0)	0 (0)	1 (0.1)	1 (0.1)	4 (0.4)	35 (3.9)	75 (8.3)	188 (20.8)	378 (41.8)	222 (24.6)	904
**Pays de la Loire**	0 (0)	0 (0)	0 (0)	0 (0)	4 (0.9)	13 (2.9)	46 (10.4)	80 (18.1)	182 (41.3)	116 (26.3)	441
**France**	3 (0.02)	3 (0.02)	21 (0.1)	84 (0.5)	229 (1.2)	861 (4.7)	2,203 (11.9)	4,165 (22.6)	6,902 (37.4)	3,991 (21.6)	18,462

Supplementary table S2 – COVID-19-associated fatalities in patients under 60 years of age.

	**< 60 years N (%)**	**≥ 60 years N (%)**	**TOTAL**
**IHU Méditerranée Infection**	1 (2.3)	42 (97.7)	43
**AP-HM total**	4 (3.1)	126 (96.9)	130
**Auvergne – Rhône-Alpes**	61 (3.7)	1,603 (96.3)	1,664
**Bourgogne-Franche-Comté**	34 (3.4)	977 (96.6)	1,011
**Bretagne**	9 (3.6)	243 (96.4)	252
**Centre – Val de Loire**	19 (3.7)	489 (96.3)	508
**Corse**	2 (3.4)	56 (96.6)	58
**Grand-Est**	152 (4.4)	3,277 (95.6)	3,429
**Hauts-de-France**	87 (5.0)	1,651 (95.0)	1,738
**Ile-de-France**	701 (9.9)	6,388 (90.1)	7,089
**Normandie**	28 (6.7)	392 (93.3)	420
**New-Aquitaine**	19 (4.8)	379 (95.2)	398
**Occitanie**	22 (4.4)	475 (95.6)	497
**Sud**	41 (4.5)	863 (95.5)	904
**Pays de la Loire**	17 (3.9)	424 (96.1)	441
**France**	**1,201 (6.5)**	**17,261 (93.5)**	**18,462**
